# C1orf112 promotes breast cancer growth by modulating the cell cycle

**DOI:** 10.3389/fbioe.2026.1717768

**Published:** 2026-03-09

**Authors:** Yuan Fang, Yikai Zheng, Lingli Jin, Manuel A. Luis, Xiaoxuan Zhu, Liyun Yong, Dongyang Liu, Fengfeng Cai, Shasha Tang

**Affiliations:** Department of Breast Surgery, Tongji Hospital, School of Medicine, Tongji University, Shanghai, China

**Keywords:** apoptosis, breast cancer, C1ORF112, prognosis, proliferation

## Abstract

**Background:**

C1orf112 (Chromosome 1 Open Reading Frame 112) is involved in DNA damage repair, and its abnormal expression has been implicated in multiple cancers, including breast cancer. This study explores the function of C1orf112 in breast cancer through bioinformatics and experimental validation.

**Methods:**

C1orf112 expression in breast cancer was analyzed using TCGA and HPA databases. Diagnostic efficiency was assessed using receiver operating characteristic (ROC) curves, and its prognostic significance was evaluated via KM plotter and TCGA data. GO and KEGG analyses were performed to explore potential mechanisms. Experimental validation included qRT-PCR and immunohistochemistry to confirm expression levels in breast cancer cells and tissues. Function assays, including CCK-8, colony formation and flow cytometry were conducted to assess the impact of C1orf112 on cell proliferation, cycle progression, and apoptosis.

**Results:**

C1orf112 was highly expressed in breast cancer, with bioinformatics and immunohistochemical analysis confirming its upregulation in tumor tissues as its potential as a diagnostic marker. Functional enrichment analysis linked C1orf112 overexpression to cell proliferation-related pathways. Immunohistochemistry revealed associations between C1orf112 expression and ER-positive status, HER2 status, and molecular subtypes. Cellular assays demonstrated that C1orf112 promotes breast cancer proliferation by influencing cell cycle regulation, involving key molecules such as CCNB1 (cyclin B1). *In vivo* experiments further confirmed these effects.

**Conclusion:**

C1orf112 contributes to breast cancer progression in association with cell cycle pathways, making it a potential diagnostic and therapeutic target with clinical applications.

## Introduction

According to 2022 GLOBOCAN data, breast cancer is the second most commonly diagnosed cancer worldwide. In China alone, 357,000 new cases were reported, accounting for 16.6% of the global total (Sung et al., 2021). Breast cancer significantly influences clinical decision-making, particularly in prognosis assessment, treatment selection, and disease monitoring. Research displays its heterogeneous nature, with biological traits and behavior potentially evolving during treatment and disease progression (Zheng et al., 2024; Barzaman et al., 2020; Liu et al., 2022; Taylor et al., 2024).

Notably, receptor expression discrepancies between primary and metastatic lesions occur in about 33% of breast cancer patients (Yi et al., 2021), despite advances in targeted therapy and immunotherapy, a substantial proportion of patients still face poor prognosis due to the lack of specific and effective diagnostic biomarkers and therapeutic targets. Recent studies have highlighted that the limitations of traditional molecular subtyping and the scarcity of actionable targets contribute to the low clinical response rate in advanced breast cancer (Chu et al., 2025), and the dysregulation of DNA damage response (DDR) and cell cycle pathways is a core driver of breast cancer progression and therapeutic resistance (Huemer et al., 2024). This underscores the urgent need to identify novel molecules that integrate DDR and cell cycle regulation for the development of precision diagnosis and targeted therapy strategies in breast cancer.

Breast cancer pathogenesis is highly complex, involving BRCA1/2 gene mutations, TP53 alterations, impaired DNA mismatch repair, ionizing radiation damage, activation of oncogenic signals, and tumor suppressor inhibition (Takahashi et al., 2021; Matta et al., 2017). Breast cancer progression is driven by complex interplay between oncogenic signaling, metabolic reprogramming, and post-transcriptional regulation. It has been reported that natural compounds have been shown to modulate PI3K/AKT/mTOR and ferroptosis pathways to inhibit cancer growth (Zhou et al., 2025), while metabolic reprogramming and chaperone-mediated autophagy promote breast cancer progression (Zhou et al., 2023). Current studies focus on exploring these mechanisms and using molecular techniques to inhibit oncogenic factors or deliver tumor-suppressing agents (Subramanyan et al., 2024). However, traditional molecular subtyping has limitations, necessitating multi-omics approaches for better classification and treatment strategies.

C1orf112 (Chromosome 1 Open Reading Frame 112) is an alpha-helical protein with well-documented roles in DNA damage response (DDR) pathways, including Fanconi anemia (FA) and homologous recombination (HR), which key mechanisms maintaining genomic stability (Stok et al., 2023; Edogbanya et al., 2021; Hashimoto et al., 2016). It is co-expressed with core DDR genes such as BRCA1, BRCA2, FANCD2, and FANCI (Stok et al., 2023; Wang et al., 2020; Evers and Jonkers, 2006), and its dysregulation is frequent in TP53-mutant tumors (Huang et al., 2020), which are common in breast cancer (Takahashi et al., 2021). Beyond DDR, emerging evidence suggests C1orf112 interacts with cell cycle regulators. Edogbanya et al. (2021) proposed it modulates meiotic recombination and cell cycle checkpoints, while Mazouzi et al. (2023) demonstrated its role in resolving HR intermediates during DNA interstrand crosslink repair, processes tightly linked to cell cycle progression. In 2012, Van Dam et al. identified a co-expression link between the mouse *BC055324* gene (human *C1orf112* homolog) and cancer-associated genes, including *RAD51* and *CCDC6* (Lauri et al., 2020). Although C1orf112 dysregulation has been noted in gastric cancer, its biological and clinical significance in cancer remains largely unexplored (Huang et al., 2020).

Notably, C1orf112s oncogenic potential has been hinted at in other cancers. Huang et al. (2020) identified it as a key gene controlling cancer stem cell characteristics in gastric cancer, and Chen et al. (2021) reported its association with poor prognosis across multiple tumor types. However, its biological and clinical significance in breast cancer remained unexplored. Given breast cancer’s reliance on DDR defects and cell cycle dysregulation for progression (Matta et al., 2017; Zhou et al., 2023), and C1orf112s dual involvement in these pathways, we hypothesized it may act as a critical oncogenic factor in breast cancer. This study was therefore designed to fill this gap by investigating C1orf112s expression, functional role, and molecular mechanisms in breast cancer.

Given its potential interactions with cell cycle regulators, C1orf112 may contribute to oncogenesis (Edogbanya et al., 2021). This study aims to investigate its expression, functional role, and molecular pathways in breast cancer through experimental and biotechnological methods.

## Materials and methods

### Data collection and processing

Breast invasive carcinoma (BRCA) expression data and corresponding clinical information were retrieved from the Cancer Genome Atlas (TCGA) database (Wu et al., 2021). Additionally, the TCGA-GTEx dataset, which integrates TCGA tumor samples and normal tissue samples, was obtained from the UCSC Xena platform (https://xenabrowser.net/datapages/). Immunohistochemical (IHC) images of normal and cancerous human tissues were sourced from the Human Protein Atlas (HPA, https://www.proteinatlas.org/). The Gene Expression Profiling Interactive Analysis (GEPIA2, http://gepia2.cancer-pku.cn) database was used to identify genes co-expressed with C1orf112 in the TCGA-BRCA dataset. Specifically, the top 100 genes positively correlated with C1orf112 were selected based on the Pearson correlation coefficient. The significance threshold for inclusion was set at a nominal p-value <0.05. For subsequent Gene Ontology (GO) and Kyoto Encyclopedia of Genes and Genomes (KEGG) pathway enrichment analyses of these co-expressed genes, the significance was defined by a false discovery rate (FDR) adjusted p-value <0.05. The cohort was dichotomized into “C1orf112-high” and “C1orf112-low” expression groups using the median expression value of C1orf112 across all samples for enrichment analysis. Since the data were obtained from publicly available sources (TCGA and UCSC Xena), this study did not require ethical approval or patient consent.

### Clinical significance of C1orf112 expression in breast cancer

To evaluate the diagnostic potential of C1orf112 in breast cancer, a ROC curve was constructed to assess the diagnostic value of C1orf112. The analysis utilized C1orf112 expression data from the TCGA-BRCA cohort and the GTEx database. The analysis was performed in R using the ‘pROC’ package. The Area Under the Curve (AUC) was computed as a measure of separability (ROBIN et al., 2011). The association between C1orf112 expression and breast cancer prognosis was assessed using the Kaplan-Meier (KM) plotter (http://kmplot.com), which employs an auto-selected optimal cutoff value to stratify patients and provides hazard ratios (HRs) with 95% confidence intervals (CIs) based on the log-rank test. Additionally, an independent survival analysis was performed on the TCGA-BRCA cohort. For this validation, patients were stratified by the median expression of C1orf112, and statistical significance was evaluated using the log-rank test via the maxstats and survmine packages in R (Tang et al., 2022).

### Tissue samples

This study analyzed 40 paired breast cancer and adjacent non-cancerous tissue samples collected from patients undergoing surgery at the Department of Breast Surgery, Tongji Hospital, School of Medicine, Tongji University (Shanghai, China). The study adhered to the Declaration of Helsinki and received approval from the Ethics Committee of Shanghai Tongji Hospital. All participants provided verbal informed consent via phone. After surgical excision, the tissues were flash-frozen in liquid nitrogen and stored at −80 °C until further analysis.

### Immunohistochemistry (IHC) analysis

IHC staining for C1orf112 was performed on 40 human breast cancer tissue samples. Briefly, paraffin-embedded tissue sections were dewaxed with xylene and rehydrated through a graded ethanol series. Endogenous peroxidase activity was blocked using 3% H_2_O_2_ for 10 min, followed by antigen retrieval via microwave heating. After cooling to room temperature, the sections were blocked with 10% fetal bovine serum for 30 min and incubated overnight at 4 °C with a primary antibody against C1orf112 (PA5-55082, Invitrogen, United States). The sections were then incubated with a universal horseradish peroxidase (HRP)-conjugated secondary antibody for both mouse and rabbit and protected from light. Immunostaining was visualized using diaminobenzidine (DAB), followed by counterstaining with hematoxylin, dehydration, and mounting.

### Immunohistochemical scoring

Two independent pathologists, blinded to clinical data, evaluated C1orf112 expression by examining the percentage of positive cells and the staining intensity. The percentage of positive cells was categorized into five grades: 0 (0%–5%), 1 (6%–25%), 2 (26%–50%), 3 (51%–75%), and 4 (76%–100%). Staining intensity was graded as 1 (weak), 2 (moderate), and 3 (strong). The final IHC score was calculated as:

IHC Score = Percentage of Positive Cells x Staining Intensity. Based on a median IHC score of 4, patients were stratified into low, medium, and high expression groups.

### Cell culture

Human breast cancer cell lines (MCF7, MDA-MB-231, MDA-MB-453, MDA-MB-468, BT549, and SKBR3) and the normal breast epithelial cell line MCF10A were obtained from the Chinese Academy of Sciences in Shanghai, China. MCF7, MDA-MB-231, MDA-MB-453, MDA-MB-468, BT549, and SKBR3 were cultured in Dulbecco’s Modified Eagle Medium (DMEM, high glucose) (Gibco, United States). MCF10A cells were maintained in RPMI-1640 medium (Gibco, United States) supplemented with mammary epithelial cell medium (Procell, Wuhan, China). All media were supplemented with 10% fetal bovine serum (Gibco, United States), 100 U/mL penicillin (Servicebio, Wuhan, China), and 100 μg/mL streptomycin (Servicebio, Wuhan, China). Cells were passaged using 0.25% trypsin-EDTA (Servicebio, China) and maintained at 37 °C with 5% CO_2_.

### Cell transfection

Small interfering RNAs (siRNAs) targeting C1orf112 (C1orf112-si1: CTC​CTC​AGT​CCT​TCA​TAT​A; C1orf112-si2: GAG​GCC​TAT​TCT​CTT​CAA​A) and the pcDNA3.1 expression vector were transfected into MDA-MB-231 and SKBR3 cells using Lipofectamine^®^ 3,000 reagent (Invitrogen, Thermo Fisher Scientific, Waltham, MA, United States). All sequences were synthesized by Generay Biotechnology (Shanghai, China).

### Colony formation assay

Transfected MDA-MB-231 and SKBR3 cells were seeded in 6-well plates at a density of 500 cells per well and cultured at 37 °C with 5% CO_2_ for 7–14 days, with medium changes every 3 days. Once colonies had formed, the medium was removed, and the cells were rinsed three times with phosphate-buffered saline (PBS, Servicebio, China), fixed in 95% ethanol for 10 min, and stained with 0.1% crystal violet (Yeasen, China). Colonies were then photographed for analysis.

### RNA extraction and cDNA synthesis

Total RNA was extracted from MCF10A, MCF7, MDA-MB-231, MDA-MB-453, MDA-MB-468, BT549, and SKBR3 cell lines, as well as from paired cancerous and para-cancerous breast tissues, using TRIzol^®^ Reagent (Invitrogen, United States). First-strand cDNA was synthesized from 1,000 ng of total RNA using the Hifair^®^ III 1st Strand cDNA Synthesis SuperMix for qPCR (Yeasen, Shanghai, China) following the manufacturer’s protocol.

### Real-time PCR

Quantitative PCR (qPCR) analysis was conducted using the LightCycler^®^ 96 Instrument (Roche Diagnostics) with TB Green Premix Ex Taq (Takara Biotechnology Co., Ltd., cat. no. RR420) to assess gene expression levels. The following primer pairs were used:

- C1orf112: Forward, 5′‐ATG​GAA​CTG​CTG​GAC​ATG​GT‐3'; Reverse, 5′‐TCA​CCC​TAG​AGT​ATG​TAT​GT‐3′
- CCNB1: forward, 5′- CAG​TTC​CGA​CTC​ATT​ATG​TCT​TCC -3′; Reverse, 5′- TCC​CTT​CTT​CTT​GTT​GCT​TCC​A -3′
- CCND1: Forward, 5′- GTC​CTA​CTT​CAA​ATG​TGT​GCA​G -3′; Reverse, 5′- GGG​ATG​GTC​TCC​TTC​ATC​TTA​G -3′
- CDK1: Forward, 5′- GGT​TCC​TAG​TAC​TGC​AAT​TCG -3′’ Reverse, 5′- TTT​GCC​AGA​AAT​TCG​TTT​GG -3′
- CDK4: Forward, 5′- ATG​TGG​AGT​GTT​GGC​TGT​ATC -3′; Reverse, 5′- CAG​CCC​AAT​CAG​GTC​AAA​GA -3′
- CDK6: Forward, 5′- GAC​CAG​CAG​CGG​ACA​AAT​A -3′; Reverse, 5′- TGA​CGA​CCA​CTG​AGG​TTA​GA -3′
- BAX: Forward, 5′- TGA​AGA​CAG​GGG​CCT​TTT​TG -3′; Reverse, 5′- AAT​TCG​CCG​GAG​ACA​CTC​G -3′
- BCL2: Forward, 5′- GTG​GAT​GAC​TGA​GTA​CCT​GAA​C -3′; Reverse, 5′- GAG​ACA​GCC​AGG​AGA​AAT​CAA -3′
- CASP3: Forward, 5′- TGG​TGA​TGA​AGG​GGT​CAT​TTA​TG -3′; Reverse, 5′- TTC​GGC​TTT​CCA​GTC​AGA​CTC -3′
- CASP9: Forward, 5′- CTG​TCT​ACG​GCA​CAG​ATG​GAT -3′; Reverse, 5′- GGG​ACT​CGT​CTT​CAG​GGG​AA
- GAPDH: Forward, 5′‐CAT​TGA​CCT​CAA​CTA​CAT​GGT​TT‐3'; Reverse, 5′‐GAA​GAT​GGT​GAT​GGG​ATT​TCC‐3′

The thermocycling conditions were as follows:

- 95 °C for 5 min
- 40 cycles of 95 °C for 10 s and 60 °C for 30 s. Relative mRNA expression levels were normalized to GAPDH using the 2^‐ΔΔCq^ method. Each experiment was performed in triplicate.

### Cell viability assay

Cell viability was assessed using the CCK-8 kit (Beyotime, China). MDA-MB-231 and SKBR3 cells were seeded in 96-well plates at a density of 2,000 cells per well and incubated overnight. At 24, 48, and 72 h, 10 µL of CCK-8 solution was added to each well, followed by incubation at 37 °C for 30, 60, 90, and 120 min. Absorbance at 450 nm was recorded using a Synergy H1 hybrid multimode microplate reader (BioTek Instruments Inc., United States) operated with Gen5 software (version 2.04).

### Cell cycle analysis and apoptosis assay

For cell cycle analysis, a Cell Cycle Detection Kit (Yeasen, China) was used. Breast cancer cells were cultured in six-well plates, collected at 80% confluence, preserved in 70% chilled ethanol for 24 h, stained with propidium iodide (PI), and analyzed using a FACS Calibur flow cytometer (BD Biosciences).

For apoptosis analysis, MDA-MB-231 and SKBR3 cells were seeded in six-well plates, transfected with siRNA or overexpression vectors, and incubated for 24 h. Cells were then collected via trypsin digestion (without EDTA) and analyzed using an Annexin V-FITC/PI kit (Yeasen, Shanghai, China) according to the manufacturer’s protocol.

### shRNA and C1orf112 overexpression lentivirus production and infection

Lentiviruses for C1orf112 knockdown (shRNA) overexpression (LV-C1orf112) were obtained from Genomeditech (Shanghai, China). Lentivirus production in HEK-293T cells was performed as follows: HEK293T cells (5 × 10^7^) were seeded in a 15-cm dish; After 16 h, PEI was used to transfect HEK-293T cells with plasmid DNA (15 µg of target plasmid, 15 µg of psPAX2, and 7.5 µg of pMD2G). The plasmid DNA was combined with 2 mL of serum-free culture medium and thoroughly mixed; 112.5 µL of PEI (1 µg/uL)was added, mixed, incubated at room temperature for 15 min, and gradually introduced to HEK-293T cells; Viral supernatants were collected 60 h post-transfection, centrifuged at 2,000 rpm for 10 min, filtered (0.45 µm), and concentrated at 15,000 rpm for a duration of 2 h at 4 °C.

### Xenograft mouse model

MDA-MB-231 cells (5 × 10^6^ cells/mL) were suspended in sterile PBS and mixed with Matrigel in equal parts. A total of 100 μL was injected into the mammary fat pads of 6-week-old anesthetized female BALB/c nu/nu mice (n = 5 per group, two groups, random assortment). Prior to the injection, mice were anesthetized using 2% isoflurane (inhaled) for the maintenance of anesthesia, ensuring a pain-free procedure. Mice were divided into 2 groups randomly. Lentiviruses were injected into tumors on days 14, 18, 22 and 26 following injection. The control group received 30 μL of Sh-NC, while the Sh-C1orf112 group received 30 μL of 1 × 10^8^ TU/mL. All mice were euthanized via carbon dioxide (CO_2_) inhalation in a 10-L sealed chamber after 1 month. The CO_2_ flow rate was controlled at 30% of the chamber volume per minute (3.0 L/min) to minimize distress. The chamber was not pre-charged with CO_2_. Instead, gas flow was initiated only after the mice were placed inside. Mice were continuously exposed to CO_2_ until the cessation of respiration and heartbeat, followed by confirmation of death via the absence of reflex responses. The tumors were collected, measured (length × width^2^/2), weighed, and analyzed via hematoxylin-eosin (H&E) and IHC staining. All animal experiments were approved by the Animal Care and Use Committee at Shanghai Tongji Hospital (No. 2024-DW-SB-023).

### Statistical analysis

Statistical analyses were performed using GraphPad Prism (version 9.0) and R software. Data are presented as mean ± SD. The choice of statistical test was based on an evaluation of normality (Shapiro-Wilk test) and homogeneity of variances (Levene’s test). Comparisons between two groups were made using an unpaired Student’s t-test (parametric) or the Mann-Whitney U test (non-parametric). For comparisons among three or more groups, one-way ANOVA followed by Tukey’s post-hoc test was used if assumptions were met; otherwise, the Kruskal–Wallis test followed by Dunn’s test with Bonferroni correction was applied. Survival data were analyzed by the log-rank test. A p-value (or adjusted p-value) of <0.05 was considered significant. *p* ≥ 0.05 (not significant), *p < 0.05, **p < 0.01, ***p < 0.001, ****p < 0.0001.

## Results

### C1orf112 is highly expressed in breast cancer

**FIGURE 1:**
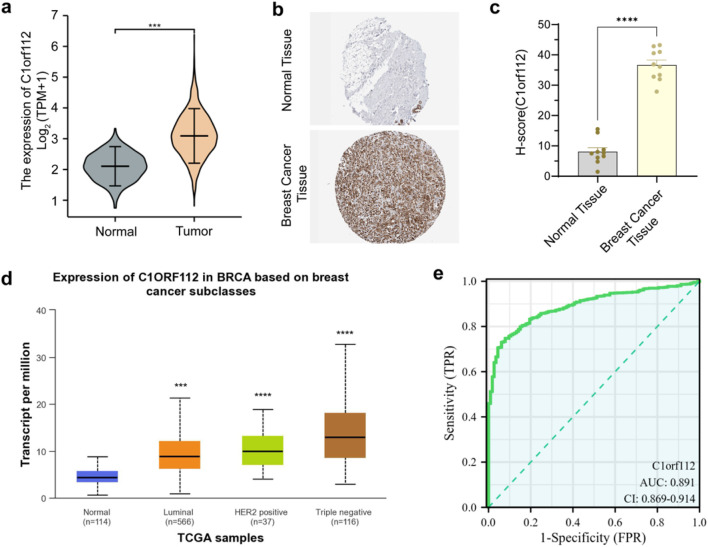
C1orf112 expression level in breast cancer from the TCGA database. **(a)** Relative expression level of C1orf112 mRNA in normal tissue and breast tumor samples. **(b)** Relative expression level of C1orf112 protein in breast cancer tissue and normal tissues from HPA. **(c)** The bar chart of relative expression level of C1orf112 protein in breast cancer tissue and normal tissues from HPA. **(d)** Relative expression level of C1orf112 in various breast cancer molecular subtypes. **(e)** ROC curves displaying the ability of C1orf112 to differentiate between breast cancer and healthy samples. *P* values were calculated by Student’s t-test; *p < 0.05, **p < 0.01, ***p < 0.001, ****p < 0.0001.

As illustrated in Figure 1, TCGA database analysis revealed that the expression level of C1orf112 was higher in breast cancer tissues compared with healthy breast tissues (Figure 1a). Consistently, IHC data from the HPA database confirmed elevated C1orf112 protein expression in breast cancer tissues relative to normal tissues (Figures 1b, c). Figure 1d illustrates the expression pattern of C1orf112 across different breast cancer molecular subtypes, with the highest expression detected in TNBC compared to Luminal (A/B) and HER2-positive subtypes. This is biologically relevant because TNBC is characterized by high genomic instability, DDR defects, and TP53 mutations (Matta et al., 2017),which pathways closely linked to C1orf112 function (Edogbanya et al., 2021). Additionally, C1orf112 exhibited high median sensitivity and specificity for breast cancer diagnosis (Figure 1e).

**FIGURE 2:**
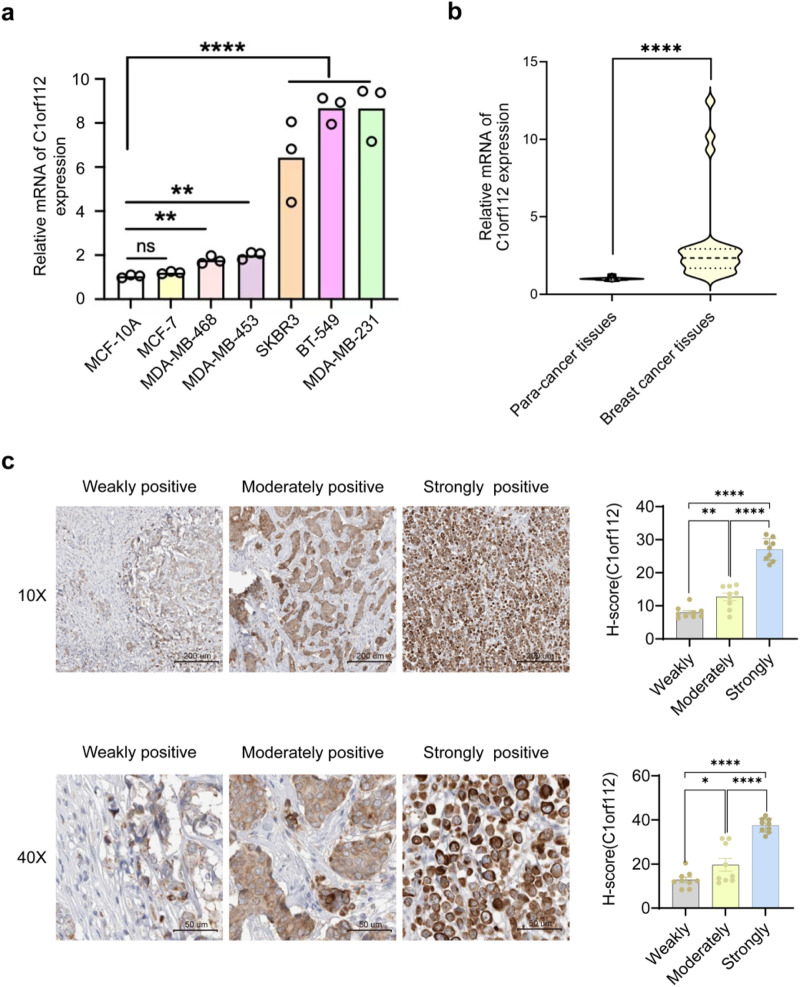
C1orf112 expression levels and representative immunohistochemical images in breast cancer tissues. **(a)** Relative expression level of C1orf112 in various breast cancer cells compared with MCF10A. **(b)** Relative expression level of C1orf112 in 40 paired tissue specimens from breast cancer patients. **(c)** C1orf112 was weakly, moderately, and strongly expressed in breast cancer tissues at ×10 and ×40 magnification. The data represent the mean ± SD; Each experiment was conducted in triplicate. *P* values were calculated by Student’s t-test; *p < 0.05, **p < 0.01, ***p < 0.001, ****p < 0.0001.

Similarly, elevated C1orf112 expression was observed in the studied breast cancer cell lines, namely, MDA-MB-231, MDA-MB-453, MDA-MB-468, BT549, and SKBR3, compared to the normal breast epithelial cell line MCF10A (Figure 2a). Among these five breast cancer cell lines, the highest mRNA expression level of C1orf112 was detected in MDA-MB-231, with elevated expression also observed in SKBR3 cells. Therefore, MDA-MB-231 and SKBR3 cell lines were selected for subsequent experiments. As expected, significantly higher C1orf112 expression levels were detected in cancerous tissues compared to matched para-cancerous tissues (Figure 2b).

To further investigate the role of C1orf112 in breast cancer, an IHC analysis of breast cancer samples was conducted. As displayed in Figure 2c, C1orf112 staining was stronger in breast cancer tissues than in para-cancer tissues. Subsequently, the staining intensity was classified into three groups—weak, moderate, and strong—to analyze its relationship with clinical characteristics. The IHC results from 40 breast cancer specimens are summarized in Table 1. Interestingly, C1orf112 expression was significantly correlated with HER2 status (*p* = 0.03). However, no significant correlations were noted between C1orf112 expression and histological type, age, or Ki67 classification in these 40 breast cancer cases. Taken together, these data suggested that C1orf112 is significantly upregulated in breast cancer tissues, supporting its potential as a subtype-specific diagnostic marker and oncogenic factor.

**TABLE 1:** Correlation between C1orf112 expression and clinicopathologic characteristics in breast cancer patients.

Characteristics	Total (n = 40)	C1orf112			*p* value
		Weak (n = 12)	Moderate (n = 13)	Strong (n = 15)	
Age, n (%)	​	​	​	​	0.188
<65 y	23 (57.5)	5 (41.7)	10 (76.9)	8 (53.3)	
>=65 y	17 (42.5)	7 (58.3)	3 (23.1)	7 (46.7)	
menopausal, n (%)	​	​	​	​	0.546
No	8 (20.0)	1 (8.3)	3 (23.1)	4 (26.7)	
Yes	32 (80.0)	11 (91.7)	10 (76.9)	11 (73.3)	
Tumor location, n (%)	​	​	​	​	0.166
Left	20 (50.0)	6 (50)	9 (69.2)	5 (33.3)	
Right	20 (50.0)	6 (50)	4 (30.8)	10 (66.7)	
Histological type, n (%)	​	​	​	​	0.602
DCIS	5 (12.5)	1 (8.3)	1 (7.7)	3 (20)	
IDC	35 (87.5)	11 (91.7)	12 (92.3)	12 (80)	
T classification, n (%)	​	​	​	​	0.441
T1	19 (47.5)	4 (33.3)	8 (61.5)	7 (46.7)	
T2	20 (50.0)	7 (58.3)	5 (38.5)	8 (53.3)	
T3	1 (2.5)	1 (8.3)	0 (0)	0 (0)	
N classification, n (%)	​	​	​	​	0.347
N0	30 (75.0)	7 (58.3)	11 (84.6)	12 (80)	
N1-N3	10 (25.0)	5 (41.7)	2 (15.4)	3 (20)	
ER status, n (%)	​	​	​	​	**0.013**
Negative	13 (32.5)	1 (8.3)	3 (23.1)	9 (60)	
Positive	27 (67.5)	11 (91.7)	10 (76.9)	6 (40)	
PR status, n (%)	​	​	​	​	0.129
Negative	13 (32.5)	2 (16.7)	3 (23.1)	8 (53.3)	
Positive	27 (67.5)	10 (83.3)	10 (76.9)	7 (46.7)	
HER2 status, n (%)	​	​	​	​	**0.037**
Negative	14 (35.0)	7 (58.3)	6 (46.2)	1 (6.7)	
Low expression	14 (35.0)	3 (25)	5 (38.5)	6 (40)	
Positive	12 (30.0)	2 (16.7)	2 (15.4)	8 (53.3)	
Molecular classification, n (%)		​	​	​	**0.046**
luminal A	15 (37.5)	5 (41.7)	7 (53.8)	3 (20)	
luminal B	5 (12.5)	4 (33.3)	1 (7.7)	0 (0)	
HER2-enriched	12 (30.0)	2 (16.7)	2 (15.4)	8 (53.3)	
Triple negative	8 (20.0)	1 (8.3)	3 (23.1)	4 (26.7)	
ki67 classification, n (%)	​	​	​	​	0.922
<= 14%	15 (37.5)	5 (41.7)	5 (38.5)	5 (33.3)	
> 14%	25 (62.5)	7 (58.3)	8 (61.5)	10 (66.7)	

The bold values indicates p < 0.05, which is statistically significant.

### C1orf112 is a candidate tumorigenic factor associated with poor prognosis in breast cancer patients

**FIGURE 3:**
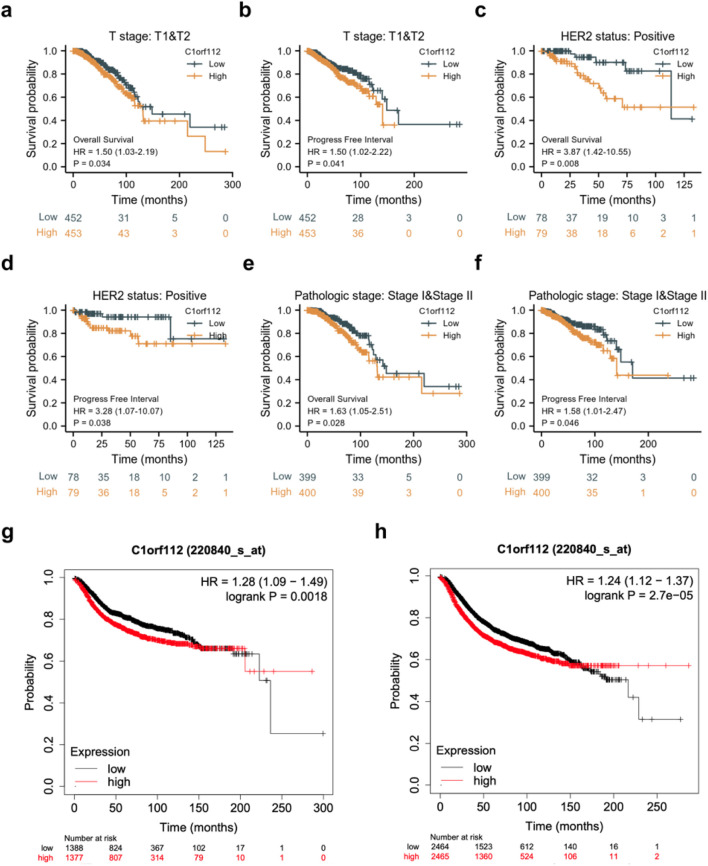
Association between C1orf112 expression and breast cancer prognosis. Kaplan–Meier curves comparing overall survival (OS) in the C1orf112 high and low groups of breast cancer patients. **(a)** OS in T stage (T1–T2); **(b)** PFS in T stage (T1–T2); **(c)** OS in HER2 status; **(d)** PFS in HER2 status; **(e)** OS in pathologic stage I & stage I; **(f)** PFS in pathologic stage I & stage II; **(g,h)** DMFS and RFS between high and low C1orf112 with breast cancer patients.

TCGA database analysis revealed that high C1orf112 expression was significantly associated with shorter OS and PFS in breast cancer patients with T1 & T2 stage (HR = 1.50, *p* = 0.034; HR = 1.50, *p* = 0.041, respectively), HER2-positive status (HR = 3.87, *p* = 0.008; HR = 3.28, *p* = 0.038, respectively), and pathologic I&II stage (HR = 1.63, *p* = 0.028; HR = 1.58, *p* = 0.046, respectively) (Figures 3a–f).

Additionally, breast cancer patients overexpressing C1orf112 exhibited shorter distant metastasis-free survival (DMFS) and relapse-free survival (RFS) compared to those with low C1orf112 levels (Figures 3g,h). These results collectively suggest that C1orf112 may serve as a potential biomarker for breast cancer prognosis.

### Loss and gain function of C1orf112 influence breast cancer proliferation *in vitro*

**FIGURE 4:**
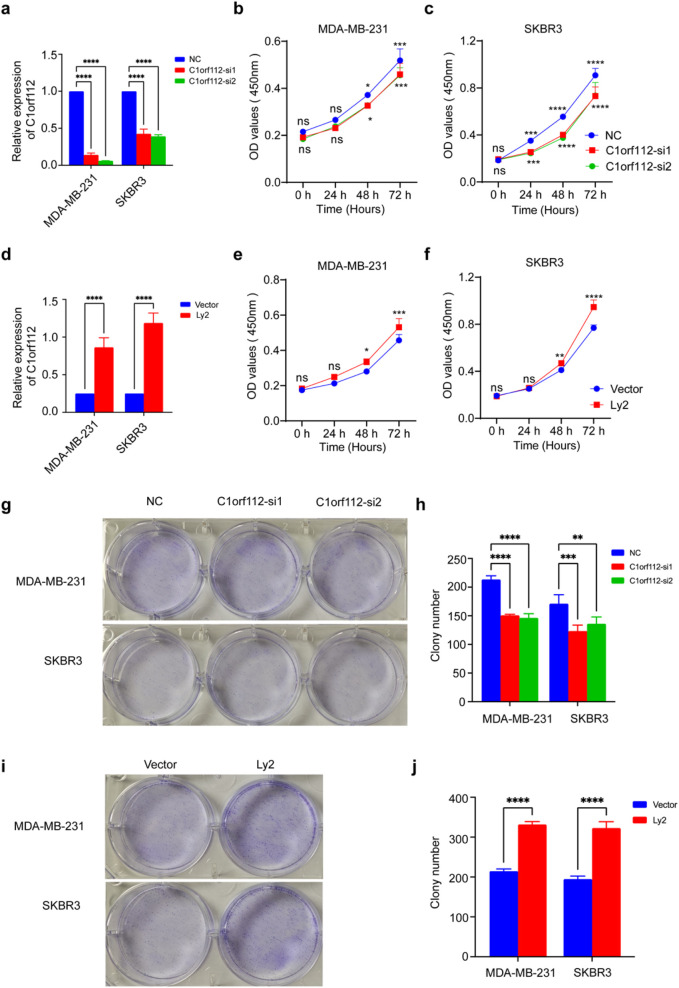
C1orf112 modulates the proliferative ability of breast cancer cells. **(a)** The effect of *C1orf112* knockdown was verified by qPCR. **(b,c)** CCK-8 cell viability assay following C1orf112 downregulation in breast cancer cell lines MDA-MB-231 and SKBR3, respectively. **(d)** The effect of *C1orf112* overexpression was verified by qPCR. **(e,f)** CCK-8 cell viability assay following *C1orf112* overexpression in breast cancer cell lines MDA-MB-231 and SKBR3. **(g,h)** Effect of *C1orf112* downregulation on colony formation. **(i,j)** Effect of *C1orf112* overexpression on colony formation. Each experiment was conducted in triplicate, and the results are presented as the mean ± standard deviation (SD). The data **(a,d)** were analyzed using the 2−ΔΔCq method with Roche LightCycler® 96 Instrument software. The data **(b,c,e,f)** was measured with BioTek Synergy H1 Gen5 software (v2.04). Colony formation images **(g,i)** were photographed using camera. Graphs **(h,j)** were generated with GraphPad Prism (v9.0). Each experiment was conducted in triplicate, and results are presented as mean ± SD. Significance levels: *p < 0.05, **p < 0.01, ***p < 0.001, ****p < 0.0001.

**FIGURE 5:**
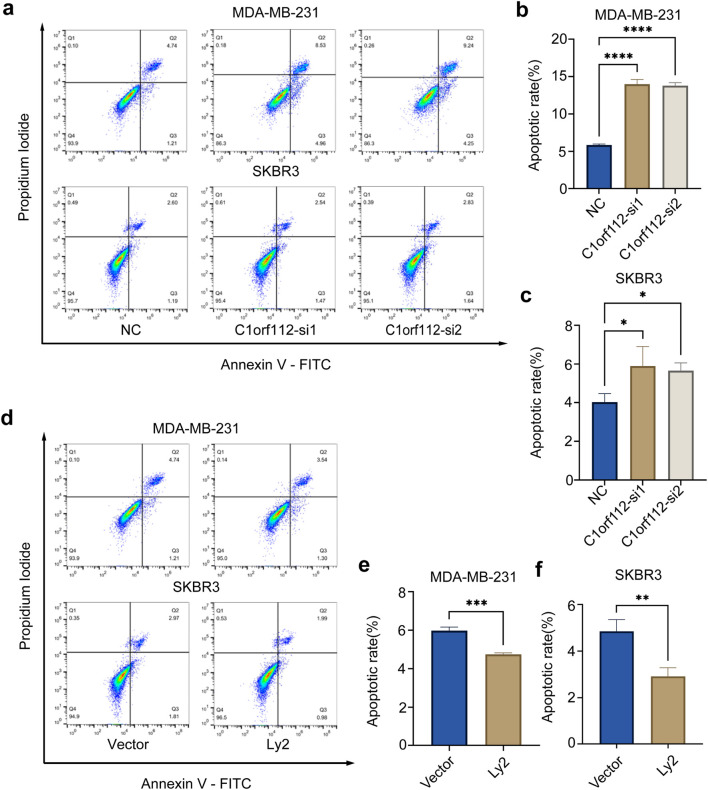
C1orf112 regulates cell apoptosis in breast cancer cells. **(a–c)** *C1orf112* knockdown enhances cellular apoptosis in breast cancer cells. **(d–f)** *C1orf112* overexpression inhibits cell apoptosis in breast cancer cells. Each experiment was conducted in triplicate. Significance levels: *p < 0.05, **p < 0.01, ***p < 0.001, ****p < 0.0001.

To further clarify the role of C1orf112 in breast cancer, a range of functional assays were performed. C1orf112 siRNAs were introduced into MDA-MB-231 and SKBR3 cell lines. To minimize off-target effects, two siRNA sequences (*C1orf112*-si1 and *C1orf112*-s2) were used. The knockdown efficiency of *C1orf112*-si1 and *C1orf112*-si2 was validated via real-time PCR (Figure 4a). Following *C1orf112* knockdown, changes in the proliferative ability of breast cancer cells were detected using CCK8, and colony formation assays. The CCK-8 assay revealed a significant reduction in breast cancer cell growth following *C1orf112* silencing (Figures 4b,c). At the same time, lentiviral transduction was used to upregulate *C1orf112* expression in MDA-MB-231 and SKBR3 cell lines. Overexpression efficiency was confirmed via real-time PCR, as shown in Figure 4d. *C1orf112* expression was significantly increased in the overexpression group (LV-C1orf112) compared to the empty vector control (LV-NC). The CCK-8 assay demonstrated that the proliferative capabilities of breast cancer cells were significantly enhanced following C1orf112 overexpression (Figures 4e,f). Colony formation assays further confirmed that colony formation was significantly suppressed upon C1orf112 depletion via siRNA (Figures 4g,h). Conversely, *C1orf112* overexpression promoted colony formation (Figures 4i,j). Additionally, flow cytometry analysis showed that C1orf112 depletion enhances cellular apoptosis, there was significant difference in the percentage of Annexin V-positive cells between the C1orf112-si and control groups (Figures 5a–c). LV-C1orf112 inhibited cell apoptosis, further promoting cell proliferation (Figures 5d–f). As expected, these results confirmed that C1orf112 modulates breast cancer cell proliferation through its pro-oncogenic function.

These functional assays demonstrate that C1orf112 acts as an oncogenic factor, knockdown inhibits cell viability and colony formation, while overexpression enhances these phenotypes. Combined with apoptosis data (Figure 5), these results confirm C1orf112 promotes breast cancer cell proliferation by suppressing apoptosis and enhancing clonogenic potential.

### C1orf112 promotes breast cancer cell proliferation by altering cell cycle progression

**FIGURE 6:**
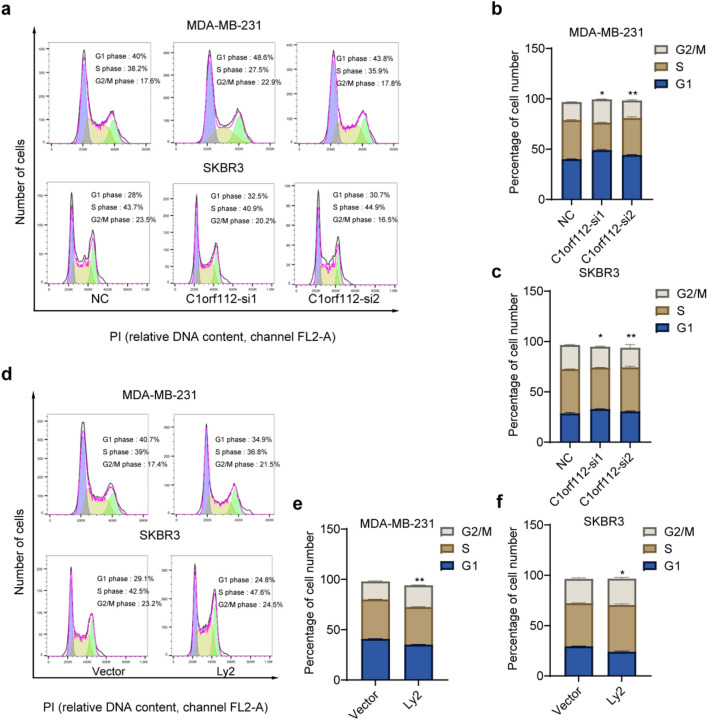
C1orf112 regulates cell cycles in breast cancer cells. **(a–c)** Effect of *C1orf112* downregulation on cell cycle distribution in breast cancer cell lines MDA-MB-231 and SKBR3. **(d–f)** Effect of *C1orf112* overexpression on cell cycle distribution in breast cancer cell lines MDA-MB-231 and SKBR3. Each experiment was conducted in triplicate. Significance levels: *p < 0.05, **p < 0.01, ***p < 0.001, ****p < 0.0001.

**FIGURE 7:**
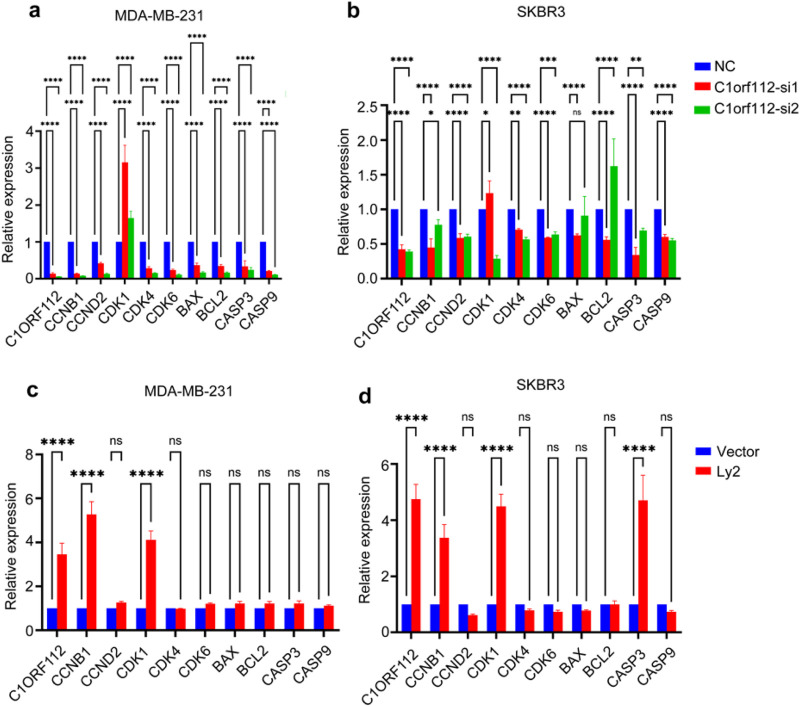
Cell cycle pathways are regulated by C1orf112. **(a,b)** The impact of *C1orf112* knockdown on cell cycle and apoptosis-related genes in breast cancer cell lines MDA-MB-231 and SKBR3. **(c,d)** The impact of *C1orf112* overexpression on cell cycle and apoptosis-related genes in breast cancer cell lines MDA-MB-231 and SKBR3. The data represent the mean ± SD; Each experiment was conducted in triplicate. *P* values were calculated by Student’s t-test; n. s not significant, *p < 0.05, **p < 0.01, ***p < 0.001, ****p < 0.0001.

Programmed cell death and dysregulation of cell cycle control are primary factors contributing to abnormal cell growth. In the present study, both knockdown and overexpression of *C1orf112* influenced cell apoptosis. To further assess the effect of *C1orf112* on cell cycle progression in MDA-MB-231 and SKBR3, flow cytometry assays (PI staining) and Western blot analyses were performed. Silencing *C1orf112* resulted in a cell cycle arrest at the G2/M phase and a concomitant reduction in the S phase (Figures 6a–c). Conversely, overexpression of C1orf112 significantly accelerated cell cycle progression (Figures 6d–f). To further understand the underlying mechanisms, the mRNA levels of key cell cycle regulators were examined. As illustrated in Figures 7a,b, depletion of C1orf112 in MDA-MB-231 and SKBR3 cells led to reduced expression of CCNB1, CCND2, CDK4, CDK6, as well as apoptosis-related genes (BAX, BCL2, CASP3, CASP9). Conversely, overexpression of *C1orf112* upregulated these genes, including *CCNB1* in MDA-MB-231 cells and both CCNB1 and Caspase3 in SKBR3 cells (Figures 7c,d). However, these findings were only detected at the mRNA level. mRNA changes may not result in functional alterations in apoptosis, the results could be validated at the protein level in future studies.

**FIGURE 8:**
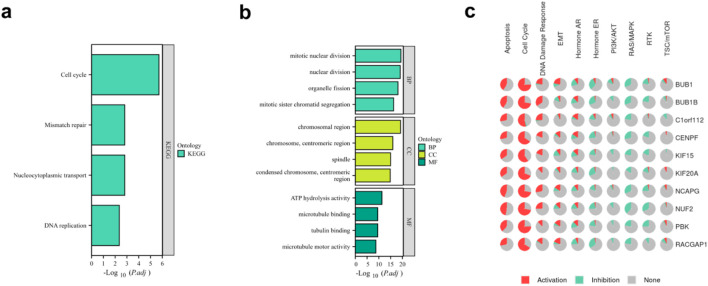
Functional enrichment analysis between high and low C1orf112 expression groups. **(a)** KEGG pathway analysis of genes coexpressed with *C1orf112*. **(b)** GO enrichment analysis identified the BP, CC, and MF terms enriched by TCGA datasets. **(c)** GSCALite analysis enriched by the genes coexpressed with *C1orf112*.

GO enrichment and KEGG pathway analyses further demonstrated that the cell cycle pathway and mitotic nuclear division were significantly enriched in the *C1orf112*-overexpressing group compared to the knockdown group (Figures 8a,b). Additionally, GSCALite (http://bioinfo.life.hust.edu.cn/web/GSCALite/) analysis confirmed a strong correlation between *C1orf112* expression and cell cycle regulation (Figure 8c), supporting the findings above. Collectively, these results indicate that cellular assays demonstrated that C1orf112 promotes breast cancer proliferation, with concurrent changes in the expression of cell cycle regulators such as CCNB1, suggesting a potential association with cell cycle modulation.

### C1orf112 promotes breast cancer growth *in vivo*

**FIGURE 9:**
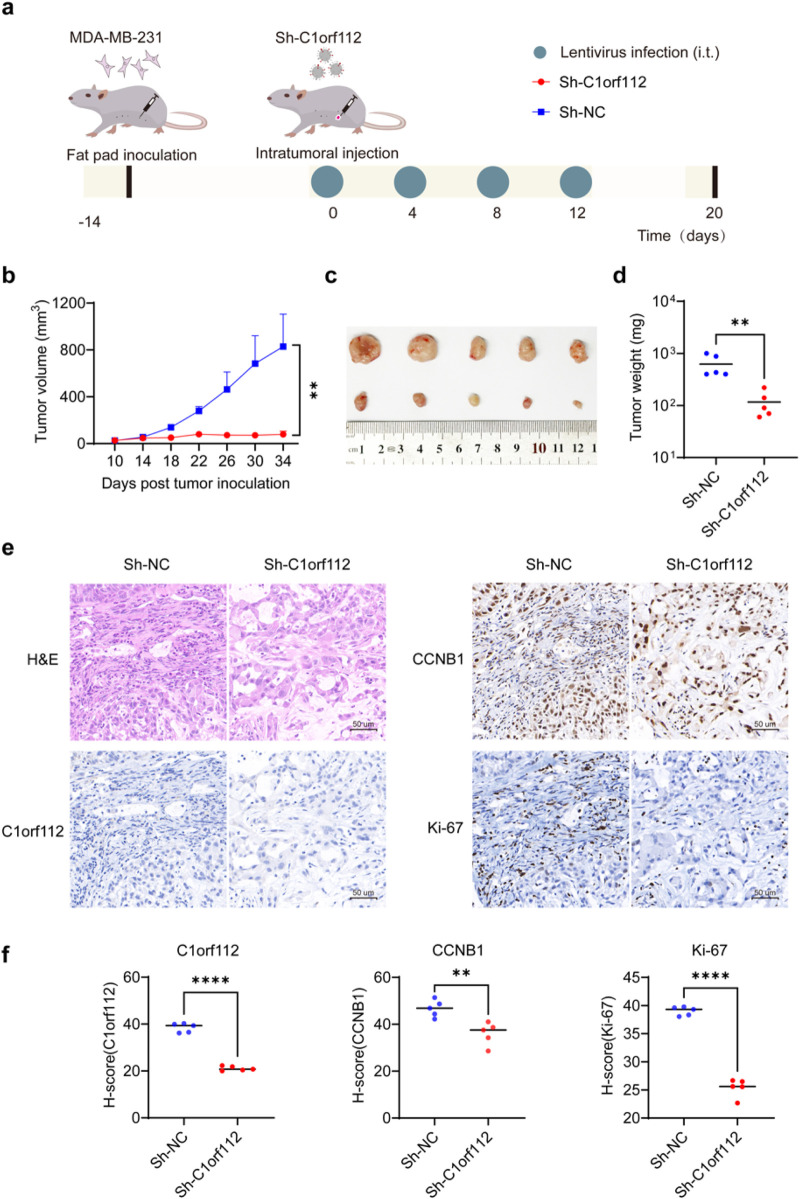
C1orf112 promotes the growth of breast cancer *in vivo*. **(a)** Flow chart of tumor bearing and virus treatment in nude mice. **(b)** Images of primary xenograft tumors derived from nude mice in Sh-C1orf112 and Sh-NC groups. **(c)** Weight of primary xenograft tumor after dissection. **(d)** Growth curve of primary xenograft tumor. **(e)** Typical images of primary xenograft tumor for HE staining, C1orf112, CCNB1 and Ki-67 in Sh-C1orf112 and Sh-NC groups. **(f)** Quantifications of C1orf112, CCNB1 and Ki-67 staining intensity in primary xenograft tumor. All staining was immunohistochemical (scale bar, 100 μm). The data **(b,d,f)** represent means ± SD. Significance levels are denoted as follows: *p < 0.05, **p < 0.01, ***p < 0.001, ****p < 0.0001.

To validate the *in vitro* findings, a xenograft mouse model was established by injecting MDA-MB-231 cells into the right primary breast fat pad of nude mice. Once tumors reached a volume of 50–100 mm^3^, the tumor-bearing mice were randomly assigned to two groups: the experimental group (Sh-C1orf112) and the control group (Sh-NC). Intratumoral viral injections were administered every 4 days for a total of four treatments (Figure 9a). Tumor size was monitored throughout the experiment.

As shown in Figures 9b–d, tumor volume and weight were significantly lower in the Sh-C1orf112 group compared to the control group. Additionally, H&E staining confirmed histopathological features of the tumor tissues (Figure 9e). IHC analysis revealed decreased expression of C1orf112 and CCNB1, as well as a lower proportion of Ki-67-positive cells in C1orf112 knockdown tumors compared to the control group (Figures 9e,f). Overall, these findings indicate that C1orf112 promotes breast cancer progression *in vivo* by modulating the cell cycle pathway.

## Discussion

This study provides comprehensive evidence that C1orf112 is a novel oncogenic factor in breast cancer, which C1orf112 is highly expressed in breast cancer tissues and cell lines and high C1orf112 expression correlates with poor prognosis in breast cancer patients. In addition, C1orf112 promotes breast cancer cell proliferation *in vitro* assays and *in vivo* xenograft model, and it modulates cell cycle progression by regulating G2/M transition and upregulating key cell cycle genes (CCNB1, CDK4, CDK6).

Although C1orf112 has been detected in various cancers, its function in breast cancer remains poorly understood. The present study elucidated the role and mechanism of C1orf112 in breast cancer, highlighting the potential as both a diagnostic marker and a therapeutic target. C1orf112 levels were elevated in breast cancer cells and tissue samples, and this increase was associated with a worse prognosis for breast cancer patients, consistent with previous research on other cancer types (Chen et al., 2021). Additionally, elevated C1orf112 levels correlated with the activation of genes that promote cell proliferation, facilitating breast cancer progression through cell cycle regulation. These findings suggest that C1orf112 may serve as an independent prognostic marker for breast cancer.

Herein, C1orf112 was found to regulate tumor growth, apoptosis, and migration in breast cancer cell lines *in vitro*. These results were further supported by *in vivo* experiments, demonstrating that C1orf112 modulated tumor growth by regulating the cell cycle, consistent with previous research (Mazouzi et al., 2023). Notably, significant differences were observed in the levels of Ki-67, a biomarker for tumor proliferation, and CCNB1, a cell cycle regulator, in primary xenograft tumors. Cell cycle dysregulation is a hallmark of cancer, and G2/M transition is a critical checkpoint for genomic stability (Martinez-Alonso and Malumbres, 2020). Our data show that C1orf112 knockdown induces G2/M arrest (Figures 6a–c), while overexpression accelerates cell cycle progression (Figures 6d–f), which consistent with its role in DDR pathways (Hashimoto et al., 2016; Mazouzi et al., 2023). CCNB1 is a key regulator of G2/M transition, forming a complex with CDK1 to drive mitotic entry (Martinez-Alonso and Malumbres, 2020). We found C1orf112 directly regulates CCNB1 expression (Figure 7), suggesting a potential mechanism that C1orf112 upregulates CCNB1 to promote G2/M progression, allowing cancer cells to bypass DDR checkpoints and proliferate despite genomic instability. This aligns with Mazouzi et al.’s (Mazouzi et al., 2023) finding that C1orf112 resolves HR intermediates and these functions enable cancer cells to survive DNA damage and proliferate uncontrollably. Collectively, our findings show that C1orf112 overexpression correlates with upregulation of CCNB1 and other cell cycle-related genes, supporting an association between C1orf112 and cell cycle progression. Future studies will be needed to validate direct regulatory mechanisms, such as protein-protein interactions or transcriptional control.

The potential involvement of C1orf112 in tumorigenesis is an area of active investigation. Increasing evidence suggests that C1orf112 may contribute to genomic stability, cell proliferation, and apoptosis regulation (Zhang et al., 2024). Disruptions in these processes can lead to uncontrolled cell growth, ultimately resulting in tumorigenesis. Previous studies have suggested that abnormal C1orf112 expression may promote oncogenesis, the process by which normal cells transform into cancerous cells. A key aspect of its function appears to be related to DNA repair. Since genomic integrity relies on efficient DNA repair mechanisms to correct replication errors (Mazouzi et al., 2023; Fernandes et al., 2018), C1orf112 may play a role in these pathways (Chen et al., 2021; Huang et al., 2021). It has been hypothesized that C1orf112 interacts with other DNA repair proteins, though the precise nature of these interactions remains unclear. Additionally, C1orf112 is hypothesized to affect meiotic recombination and regulation of cell cycle checkpoints (Edogbanya et al., 2021; Fernandes et al., 2018), which are crucial control mechanisms that prevent cells from progressing through the cell cycle with damaged DNA (Martinez-Alonso and Malumbres, 2020; KING and Cidlowski, 1980). Dysregulation of these checkpoints can lead to an increased risk of mutations and chromosomal instability, both of which are hallmarks of cancer cells (Shorrocks et al., 2004; Li et al., 2024).

Our findings align with growing evidence that cell cycle regulation is intertwined with metabolic reprogramming (Zhou et al., 2023) and growth factor signaling in breast cancer, while VEGF-FGF signaling activates quiescent stem cells to drive proliferation (Chen et al., 2024). Future studies could explore whether C1orf112 interacts with these pathways to modulate cell cycle progression. In addition, Overexpression of C1orf112 disrupts these tightly regulated processes, accelerating the transition through cell cycle phases. Specifically, increased C1orf112 levels correlated with a higher proportion of cells transitioning from the G1 phase to the S phase, where DNA replication occurs. This shift reflects an increased number of cells actively engaged in DNA replication (Ekundayo and Bleichert, 2019; Méchali, 2001). Moreover, the increased presence of cells in the G2/M phase suggests that they are rapidly progressing through the S phase and preparing for mitosis. Flow cytometry analysis has corroborated these findings, demonstrating that *C1orf112*-overexpressing cells exhibit a reduced G1 population and an increased proportion of cells in the S and G2/M phases. Conversely, control cells display a more balanced distribution across cell cycle phases. These results are consistent with previous studies, reinforcing the role of C1orf112 in cell cycle regulation (Mazouzi et al., 2023; Raschle et al., 2015).

A C1orf112 has not been extensively characterized, it has traditionally been recognized as a nuclear protein involved in DNA replication and DNA damage responses (Zhang et al., 2024). Previous research has demonstrated that high C1orf112 expression is significantly associated with shorter progression-free survival and overall survival in various cancers, particularly in sarcoma patients (Chen et al., 2021). Zhang et al. were the first to reveal that C1orf112 functions beyond nuclear DNA replication, influencing mitochondrial activity and thereby affecting the growth and metastasis of osteosarcoma (Zhang et al., 2024). Their study demonstrated that C1orf112 protein levels are positively correlated with methionine concentration in osteosarcoma cells, suggesting that methionine availability regulates C1orf112 expression. Furthermore, C1orf112 was found to be primarily localized in mitochondria rather than the nucleus. Knockdown of *C1orf112* resulted in reduced osteosarcoma cell growth and migration, as well as significantly impaired mitochondrial function, even in methionine-rich culture conditions. Whether C1orf112 is regulated at the post-transcriptional level, or modulates cell cycle genes via such mechanisms, warrants further investigation. Finally, pathway-based therapeutic strategies, such as targeting MAPK and AKT/mTOR (Kaplan and Mete, 2023), highlight the clinical potential of inhibiting cell cycle-related oncogenes like C1orf112. Additionally, post-transcriptional regulation plays a key role in breast cancer, with RNA regulators controlling cell cycle gene expression (Vignesh and Thangamalar, 2024; Hussain et al., 2024; Zhou et al., 2021) and integrating with canonical pathways such as Wnt/β-catenin (Sun et al., 2025). Whether C1orf112 is regulated at the post-transcriptional level, or modulates cell cycle genes via such mechanisms, warrants further investigation.

In summary, this study provided substantial evidence that C1orf112 modulates tumor growth in breast cancer both *in vitro and in vivo*. While our data highlight associations between C1orf112 and cell cycle gene expression, there has several limitations that protein-level validation and functional rescue experiments are required to confirm whether these relationships are causal. Thus, targeting this pathway might be a promising therapeutic strategy for the clinical management of breast cancer.

## Data Availability

The datasets presented in this study can be found in online repositories. The names of the repository/repositories and accession number(s) can be found in the article/supplementary material.
